# MlrA, a MerR family regulator in *Vibrio cholerae*, senses the anaerobic signal in the small intestine of the host to promote bacterial intestinal colonization

**DOI:** 10.1080/19490976.2022.2143216

**Published:** 2022-11-11

**Authors:** Jialin Wu, Yutao Liu, Wendi Li, Fan Li, Ruiying Liu, Hao Sun, Jingliang Qin, Xiaohui Feng, Di Huang, Bin Liu

**Affiliations:** a TEDA Institute of Biological Sciences and Biotechnology, Nankai University, Tianjin, China; b Key Laboratory of Molecular Microbiology and Technology, Nankai University, Ministry of Education, Tianjin, China; c Nankai International Advanced Research Institute, Nankai University Shenzhen, China

**Keywords:** *Vibrio cholerae*, MerR family regulator, toxin-coregulated pilus, MlrA, pathogenicity

## Abstract

*Vibrio cholerae* (*V. cholerae*), one of the most important bacterial pathogens in history, is a gram-negative motile bacterium that causes fatal pandemic disease in humans via oral ingestion of contaminated water or food. This process involves the coordinated actions of numerous regulatory factors. The MerR family regulators, which are widespread in prokaryotes, have been reported to be associated with pathogenicity. However, the role of the MerR family regulators in *V. cholerae* virulence remains unknown. Our study systematically investigated the influence of MerR family regulators on intestinal colonization of *V. cholerae* within the host. Among the five MerR family regulators, MlrA was found to significantly promote the colonization capacity of *V. cholerae* in infant mice. Furthermore, we revealed that MlrA increases bacterial intestinal colonization by directly enhancing the expression of *tcpA*, which encodes one of the most important virulence factors in *V. cholerae*, by binding to its promoter region. In addition, we revealed that during infection, *mlrA* is activated by anaerobic signals in the small intestine of the host through Fnr. In summary, our findings reveal a MlrA-mediated virulence regulation pathway that enables *V. cholerae* to sense environmental signals at the infection site to precisely activate virulence gene expression, thus providing useful insights into the pathogenic mechanisms of *V. cholerae*.

## Introduction

*Vibrio cholerae* (*V. cholerae*), a gram-negative bacterium, is the etiological agent of cholera, which affects millions of individuals leading to approximately 100,000 deaths per year.^1^ Although the development of oral rehydration therapy has dramatically reduced the fatality rate of treated cases, cholera continues to present a severe global health and economic challenge.^2^ Thus far, over 200 serogroups of *V. cholerae* have been identified. The serogroup O1 El Tor biotype, which appears to have emerged in 1961 in Indonesia, is currently the predominant cause of cholera globally.^3^

*V. cholerae* has a complex life cycle involving transitions between various aquatic environments, such as surface seawater, and the human small intestine.^4^ *V. cholerae* can survive in aquatic environments year-round.^5^ In the host, *V. cholerae* preferentially colonizes the epithelium of the distal small intestine.^6^ Once it enters the small intestine, *V. cholerae* mainly produces two major virulence factors: the cholera toxin (CT) encoded by *ctxAB* on the lysogenic CTXΦ bacteriophage, which directly causes diarrhea,^7^ and the toxin-coregulated pilus (TCP), which is required for bacterial attachment to enterocytes and intestinal colonization.^8^ TCP belongs to the type-4 Pilli family,^9^ and has been identified as a critical colonization factor for *V. cholerae* in both animal models and humans.^10^ TCP is a polymer of repeating subunits of the major pilin protein, TcpA,^11^ which is encoded by *tcpA*.^12^ In *V. cholerae*, the regulation of TCP biosynthesis is complex and orchestrated, forming an elaborate regulatory network. The TCP operon is mainly activated by the AraC/XylS-family transcriptional regulator, ToxT,^13^ which is regulated by TcpP and ToxR.^14^ Recently, TCP was also found to be regulated by other regulators, including Fur,^15^ HapR,^16^ AhpAB,^17^ CRP^18^ and CarR.^19^ However, the regulatory mechanisms underlying TCP have not been fully elucidated.

Upon transition into the gut, *V. cholerae* undergoes a carefully orchestrated set of gene expression changes to adapt to host-specific environmental stresses, such as temperature, pH, oxygen concentration, and bile salts.^20^ Among these intestinal signals, oxygen concentration is a key environmental factor that governs the physiology of *V. cholerae*. Within the small intestine, oxygen concentration decreases below 3%^21^ and is further lowered to a nearly anoxic environment by the action of the commensal microbiota and host metabolism.^22^ It has been shown that *V. cholerae* promotes its colonization capacity in response to anaerobic environment.^23^ For example, TCP induction was detected when *V. cholerae* cultures were subjected to oxygen deprivation.^24^ To adapt to the low oxygen concentration, trimethylamine N-oxide (TMAO) can substitute oxygen as the final electron acceptor, and stimulate CT production in *V. cholerae*.^25^

Members of the MerR family of regulators are widespread in bacteria and play important roles in adaptation to environmental challenges.^26^ In general, MerR family regulators consist of an N-terminal helix-turn-helix DNA binding region and a C-terminal effector binding region, which respond to environmental stimuli including metal ions.^27^ Binding of the metal ion at the C-terminal inductor-binding site of MerR family regulators provokes an allosteric change at the N-terminal DNA binding region of the protein, which in turn transduces changes in the promoter structure, resulting in transcription activation.^27,28^ MerR family regulators have been proven to affect virulence in many bacteria, such as ZntR in *Brucella* strains,^29^ VarN in *Salmonella enterica* serovar Typhimurium (*Salmonella* Typhimurium);^30^ CueR in *Pseudomonas aeruginosa*, and SoxR in *Vibrio vulnificus*.^31,32^ However, whether MerR family regulators are involved in the virulence regulation of *V. cholerae* has not been well characterized.

In this study, we aimed to systematically investigate the effects of all five annotated MerR family regulators on *V. cholerae* pathogenicity. Among these regulators, VCA0056 (MlrA) was shown to be essential for the efficient intestinal colonization of bacteria. Moreover, MlrA was activated by Fnr in response to the anaerobic signal in small intestine, and then it activated the TCP operon by directly binding to the promoter of *tcpA*. Overall, this study characterizes a novel MlrA-mediated regulatory pathway to enhance the virulence and colonization capacity of *V. cholerae*.

## Results

### Multiple MerR family regulators impact intestinal colonization in V. cholerae

**Figure 1.**
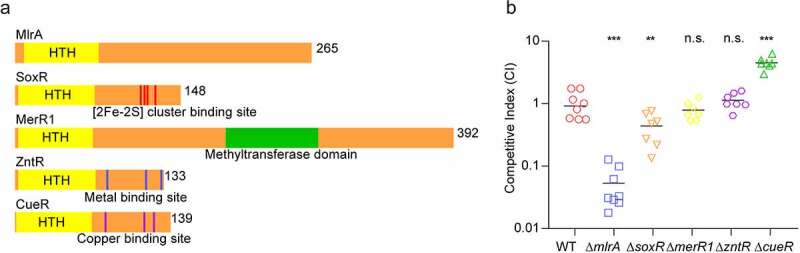
The effect MerR family regulators in the colonization of *V. cholerae.* (a) Domain structure of MlrA, SoxR, MerR1, ZntR, and CueR in *V. cholerae*. (b) Competition assay comparing the colonizing ability of WT, Δ*mlrA*, Δ*soxR*, Δ*merR1*, Δ*zntR*, and Δ*cueR* strains in the infant mouse intestine. Competitive index (CI) is defined as the output ratio of mutant strains to WT *lacZ*-, divided by the input ratio of mutant strains to WT *lacZ*-. Each symbol represents the CI in an individual mouse; horizontal bars indicate the median. A two-sided Mann–Whitney *U* test was used to calculate *P* values. * *P* < .05, ** *P* < .01, *** *P* < .001; n.s., not significant.

Homologs of MerR family regulators were screened for in the genome *of V. cholerae* EL2382, the 7^th^ pandemic strain.^33^ Five genes were annotated as MerR family regulators: *vca0056* (*mlrA), vca0084* (*soxR), vca0264* (*merR1), vc0277* (*zntR), and vc0974* (*cueR*). Domain structure analysis of these regulators revealed that all five proteins contain an N-terminal DNA-binding helix-turn-helix (HTH) motif (Figure 1a). C-terminal domain structure analysis showed that VC0277 and VC0974 contain a metal-binding site, VCA0084 contains a [2Fe-2S] cluster-binding site, and VCA0264 contains a methyltransferase domain. No predicted domains were found in VCA0056 (MlrA). This suggests that these five MerR family regulators may have different functions and perform different roles in *V. cholerae* virulence.

In-frame deletion mutants of MerR family regulators were generated to evaluate their effect on bacterial intestinal colonization. The competitive assay in infant mice showed that the competitive index (CI) values of Δ*mlrA*, Δ*soxR*, Δ*merR1*, Δ*zntR*, and Δ*cueR* strains versus wild type (WT) were 0.058, 0.439, 0.788, 1.132, and 4.161 (Figure 1b), respectively. Consistently, the competitive infection assay results exhibited trends similar to those of a previous Tn-Seq analysis in infant rabbit.^34^ These data indicate that MlrA may play an important role in the intestinal colonization of *V. cholerae*.

## Inducing the expression of *mlrA* in small intestine promotes the colonization of *V. cholerae*

**Figure 2.**
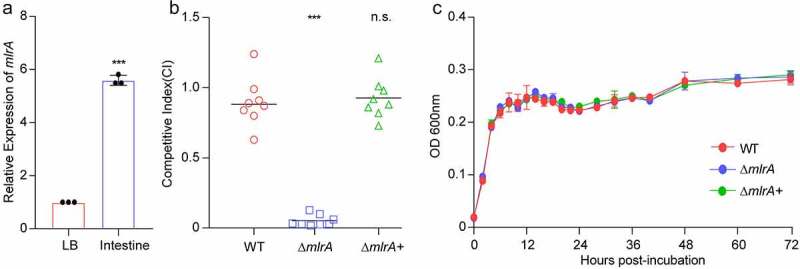
MlrA promotes colonization in *V. cholerae.* (a) qRT-PCR expression level of *mlrA* in WT in vitro and the mice small intestine. Data are represented as the mean ± SD (n = 3). A two-tailed unpaired Student’s *t*-test was used to calculate *P* values. (b) Competition assay comparing the colonizing ability of WT, Δ*mlrA*, and Δ*mlrA*+ strains in the infant mouse intestine. CI is defined as the output ratio of mutant strains to WT *lacZ*-, divided by the input ratio of mutant strains to WT *lacZ*-, or output ratio of complemented strains to WT *lacZ*- with pBAD33, divided by the input ratio of complemented strains to WT *lacZ*- with pBAD33. Each symbol represents the CI in an individual mouse; horizontal bars indicate the median. A two-sided Mann–Whitney *U* test was used to calculate *P* values. (c) Growth curves of WT, Δ*mlrA*, and Δ*mlrA*+ in LB medium under anaerobic conditions. * *P* < .05, ** *P* < .01, *** *P* < .001; n.s., not significant.

Next, we compared the expression of *mlrA* by qRT-PCR in *V. cholerae*-infected infant mouse intestine and Luria-Bertani broth (LB) media. The results revealed that *mlrA* expression was approximately 5.25-fold higher in the small intestine of mice than *in vitro* (Figure 2a). This indicated that *mlrA* may have a positive effect on the colonization of *V. cholerae in vivo*. We further analyzed the colonization ability of Δ*mlrA* and the complementary strain (Δ*mlrA*+) using a competitive assay. Consistent with the previous results, the competitive assay showed that the colonization ability of Δ*mlrA* was restored to the WT level upon complementation (Figure 2b). These results confirmed that a mutation in *mlrA* attenuated the colonization ability of *V. cholerae* in mice. As the mouse intestine is an anaerobic environment, we examined the growth curves of WT, Δ*mlrA*, and Δ*mlrA*+ under anaerobic conditions. The growth curve showed that no significant growth defect was identified among WT, Δ*mlrA*, and Δ*mlrA*+ *in vitro* (Figure 2c), indicating that the influence of MlrA on the intestinal colonization of *V. cholerae* was not due to different growth rates.

### MlrA increases bacterial virulence by regulating tcpA expression by directly binding to its promoter region

**Figure 3.**
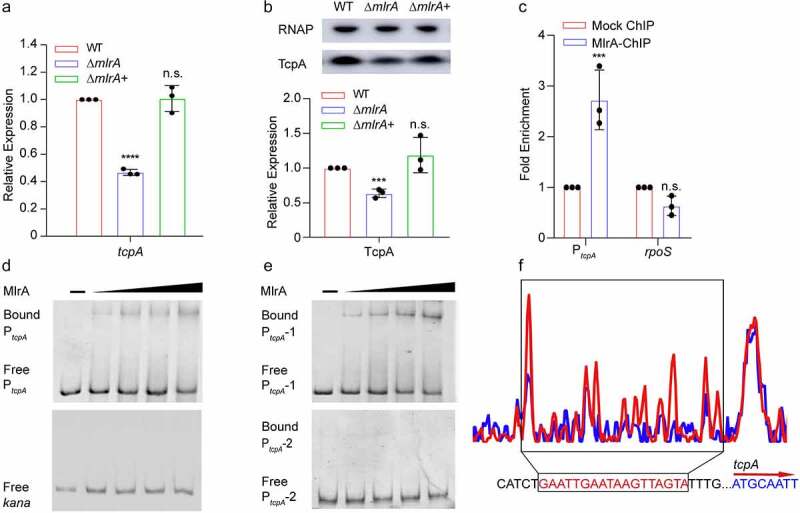
MlrA directly promotes the expression of *tcpA.* (a) qRT-PCR expression level of *tcpA* in WT, Δ*mlrA*, and Δ*mlrA*+ strains at the logarithmic phase in AKI medium. Data are represented as the mean ± SD (n = 3). (b) Representative western blotting image and quantitative analysis of TcpA in WT, Δ*mlrA* and Δ*mlrA*+ strains in AKI medium. RNA polymerase (RNAP) was used as a loading control. Data are represented as the mean ± SD (n = 3). (c) The fold enrichment of the promoters for *toxT* and the negative control (*rpoS*) in the chromatin immunoprecipitation assay. (d) EMSA of the specific binding of purified MlrA to the promoter region of *tcpA* and *kana* (negative control). (e) EMSA of the specific binding of purified MlrA to the promoter region of *tcpA* without the −10 and −35 elements (P*_tcpA_*-1), and the promoter region of *tcpA* without the binding site (P*_tcpA_*-2). (f) MlrA binds to a motif in the *tcpA* promoter region. The protected region shows a significantly reduced peak intensities (blue) pattern compared to the intensities seen in the control (red). The identified MlrA-binding motif is shown in a box at the bottom of the figure. Two-way ANOVA (a, b) and two-tailed unpaired Student’s *t*-test (C) were used to calculate *P* values. * *P* < .05, ** *P* < .01, *** *P* < .001; n.s., not significant.

To analyze the mechanism by which *mlrA* regulates the colonization of *V. cholerae*, RNA-seq of WT and Δ*mlrA* grown in AKI medium was performed. A total of 153 genes showed differential expression between WT and Δ*mlrA*, when considering p-values <0.05, and |fold-change|≥2. Overall, 46 downregulated and 107 upregulated genes were identified in the Δ*mlrA* strain (Table S1). Among these genes, the TCP operon genes were significantly downregulated in Δ*mlrA*. qRT-PCR analysis of the expression of major virulence genes in *V. cholerae*, including *tcpA, toxR, toxT, ctxA*, and *tcpP*, showed that only the expression of *tcpA*, which encodes the basic protein of TCP, was significantly decreased in Δ*mlrA* compared with WT, and was restored to wild-type levels in Δ*mlrA*+ (Figure 3a). However, the expression of *tcpP, toxR, toxT*, and *ctxA* was not significantly different among the WT, Δ*mlrA*, and Δ*mlrA*+ strains (Fig. S1A), which is consistent with the RNA-seq results (Table S1). Furthermore, western blotting revealed that the production of TcpA was reduced in Δ*mlrA* compared with that in WT, which was restored to WT levels in Δ*mlrA*+ (Figure 3b). In contrast, no difference in the production of cholera toxin was detected among WT, Δ*mlrA*, and Δ*mlrA+* (Fig. S1B). These results indicate that MlrA positively regulates the expression of the TCP operon but not other virulence genes.

Next, we investigated whether MlrA directly regulated tcpA expression by binding to its promoter. An electrophoretic mobility shift (EMSA) assay was performed using purified MlrA-His6. As shown in Figure 3d, with increasing concentrations of MlrA protein; migrating bands were observed for the promoter of *tcpA*. MlrA could not bind to the promoter region of *tcpP, toxR, toxT ctxA*, or *kana* (negative control) under the same experimental conditions (Fig. S2A and Figure 3d). Consistent with this, chromatin immunoprecipitation-qPCR (ChIP-qPCR) results showed that the promoter of *tcpA* was enriched 2.76-fold in MlrA-ChIP samples compared with mock ChIP samples (Figure 3c), which confirmed the results of EMSA. In contrast, fold enrichment of *rpoS* and the promoters of *tcpP, toxR, toxT*, and *ctxA* showed no significant difference between the MlrA-ChIP and mock ChIP samples (Figure 3c and S2B). These results indicated that MlrA specifically binds to the *tcpA* promoter region both *in vitro* and *in vivo*.

Typically, the binding site of classical MerR family regulators is always located in the spacer between the −10 and −35 elements of the promoters of downstream genes. When the C-terminal effector-recognition domain binds to its cognate ligand, the transcription factor untwists and shortens the DNA, realigning the −10 and −35 elements to allow the RNA Polymerase (RNAP) holoenzyme to bind and activate transcription.^35^ To verify whether MlrA specifically binds to the −10 and −35 elements of the *tcpA* promoter, an EMSA of P*_tcpA_*-1(−10 and −35 elements deleted in the *tcpA* promoter) was performed (Figure 3e). The results showed that MlrA could still bind to the *tcpA* promoter without the −10 and −35 elements. This indicated that the binding site of MlrA was not located between the −10 and −35 elements of the *tcpA* promoter region. To identify the precise binding site for MlrA in the *tcpA* promoter region, a dye-based DNase I footprinting assay was performed. The results revealed a specific MlrA-bound sequence containing an 18–base pair motif (5´-GAATTGAATAAGTTAGTA-3´, −234 to −217 bp from the *tcpA* translational start site) in the *tcpA* promoter region (figure 3f). To further confirm that this motif is necessary for the binding of MlrA, EMSA of P*_tcpA_*-2 (the binding site identified by the dye-based DNase I footprinting assay deleted in the *tcpA* promoter) was performed (Figure 3e). The results showed that P*_tcpA_*-2 cannot bind to MlrA, confirming that the 18 base pair motif we identified is required for the binding ability of MlrA to P*_tcpA_*.

### Activation of tcpA by MlrA is not mediated by metal ions

**Figure 4.**
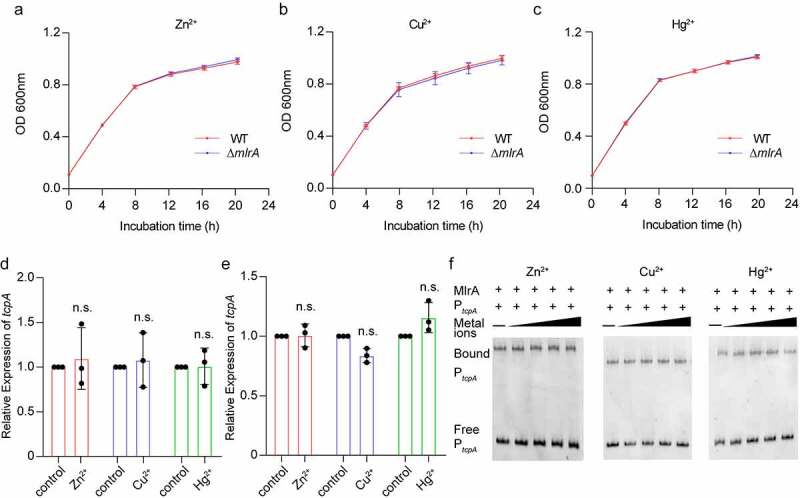
Metal ions do not affect the function of MlrA. (a-c) Growth curves of WT and Δ*mlrA* in LB medium under aerobic conditions with ZnCl_2_ (a), CuCl_2_ (b), and HgSO_4_ (c). (d, e) qRT-PCR expression level of *tcpA* in Δ*mlrA* (d), and WT (e), with or without metal ion in AKI medium. (f) The influence of ZnCl_2_, CuCl_2,_ and HgSO_4_ on the binding of purified MlrA protein to the promoter region of *tcpA*. EMSAs of 0.2 μM purified MlrA protein complexed with 40 ng of the DNA fragment in the presence of different concentrations of metal. A two-tailed unpaired Student’s *t*-test was used to calculate *P* values. * *P* < .05, ** *P* < .01, *** *P* < .001; n.s., not significant.

Members of the MerR family regulators are commonly found in different bacteria as metal sensing regulators.^36^ Therefore, we speculated that the activation of *tcpA* by MlrA also depends on metal ions. The growth curve was plotted for Δ*mlrA* and WT in LB media supplemented with ZnCl_2_, CuCl_2_, and HgSO_4_, which always interact with the MerR family regulators.^37^ The results showed that there were no differences in growth between Δ*mlrA* and WT samples under these conditions (Figure 4a-c). To investigate whether MlrA-mediated activation of *tcpA* expression is influenced by metal ions, the expression of *tcpA* in WT and Δ*mlrA* in AKI media with or without metal ions was analyzed by qRT-PCR. The results showed that the presence of metal ions did not influence the expression of *tcpA* in Δ*mlrA* or the WT (Figure 4d, e). Furthermore, EMSA confirmed that the binding of MlrA to the *tcpA* promoter was not influenced by metal ions (figure 4f). These results suggest that the regulation of the *tcpA* by MlrA is not regulated by metal ions.

### mlrA is activated by Fnr in an anaerobic environment

**Figure 5.**
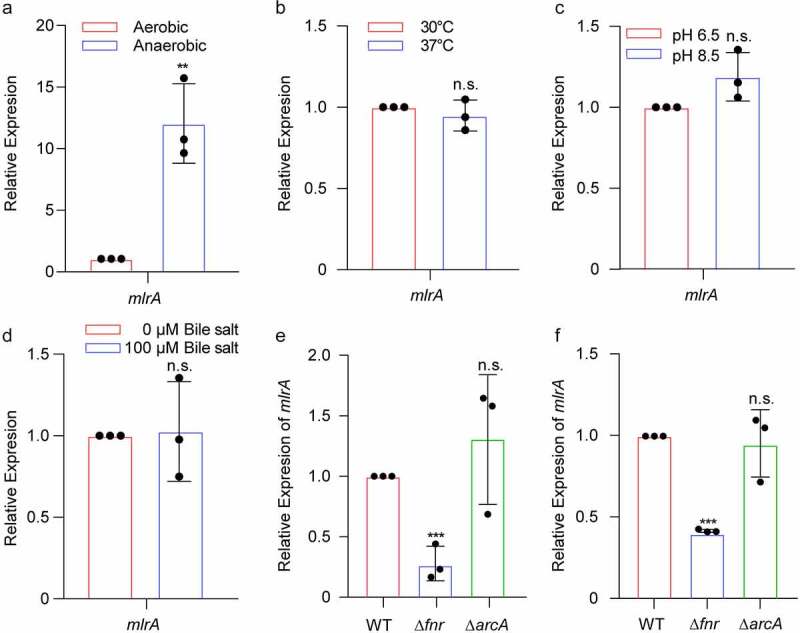
MlrA was activated by Fnr under anaerobic condition. (a-d) qRT-PCR expression levels of *mlrA* in WT under aerobic or anaerobic conditions (a), at 30°C and 37°C (b), pH 6.5 and pH 8.5 conditions (c), and with 0 μM or 100 μM bile salt (d), in LB medium. (e, f) qRT-PCR expression level of *mlrA* in WT, Δ*fnr*, and Δ*arcA* at the stationary phase in LB medium under anaerobic conditions (e), and in the small intestine of mice (f). Significance was determined by a two-tailed unpaired Student’s *t*-test (A-D) and two-way ANOVA (E, F). * *P* < .05, ** *P* < .01, *** *P* < .001; n.s., not significant.

As *mlrA* does not respond to metal ion signals, it exhibits significant upregulation in the small intestine of mice. We speculated that MlrA might respond to some unique signals *in vivo*. Temperature, pH, oxygen level, and bile salt presence are the major differing factors between the aquatic environment and the infant mouse intestine. The expression of *mlrA* under these four different conditions was compared using qRT-PCR. The results showed that the expression of *mlrA* was enhanced under anaerobic condition, but was not influenced by temperature, pH, or bile salts (Figure 5a-d). This indicates that *mlrA* expression is upregulated in an anaerobic environment.

Fnr and ArcA are the two major global regulators in response to anaerobic signals in *V. cholerae*,^38^ and qRT-PCR assays were performed to investigate whether *mlrA* expression is regulated by Fnr and/or ArcA under anaerobic conditions. The qRT-PCR results showed that the expression of *mlrA* exhibited a 3.80-fold decrease in Δ*fnr* compared to WT under anaerobic conditions (Figure 5e). However, *mlrA* expression was not significantly different between Δ*arcA* and WT under the same conditions (Figure 5e). Furthermore, *mlrA* expression in infant mouse intestines was analyzed in WT, Δ*fnr*, and Δ*arcA* variants. The results showed that the expression of *mlrA* was downregulated in Δ*fnr* compared to WT *in vivo*, while there was no significant difference in *mlrA* expression between WT and Δ*arcA in vivo* (figure 5f). These results suggest that *mlrA* expression is regulated by anaerobic signaling via Fnr *in vitro* and *in vivo*.

Taken together, these data indicate the expression of *mlrA* is activated by Fnr in response to the anaerobic signal in the small intestine of host, which leads to the increased pathogenicity of *V. cholerae* by directly enhancing the expression of *tcpA*.

## Discussion

The MerR family transcriptional regulators are widespread in bacteria and activate the transcription of genes to perform multiple functions.^39^ However, the roles of the MerR family regulators in *V. cholerae* remain unclear. In this study, a novel MerR family regulator-mediated virulence regulation mechanism was identified in *V. cholerae*. We found that the expression of *mlrA* is upregulated in the small intestine of the host, and thus promotes the colonization of bacteria by directly upregulating the expression of TCP. MlrA in *E. coli*, which shares 50% sequence similarity with MlrA in *V. cholerae*, regulates biofilm formation through the major matrix regulator CsgD.^40^ However, the RNA-seq results did not show differences between the WT and Δ*mlrA* in the expression of genes related to biofilm formation, such as biofilm formation master regulator *vpsR* and Vibrio polysaccharide (VPS) operons, indicating that the function of MlrA in *V. cholerae* differs from its homolog in *E. coli*. By analyzing the genome of *V. cholerae* EL2382, we found that there were homologous sequences of identified MlrA-binding sites in the promoter region of two genes (*vca0219* and *vc0910*). The RNA-seq data showed that the expression of *vca0219* and *vc0910* are downregulated 3.12-fold and upregulated 2.10-fold, respectively, in Δ*mlrA*, suggesting that MlrA may directly regulate the expression of these two genes. *Vca0219* encodes the hemolysin (HlyA),^41^ which is an important virulence factor in *V. cholerae* that belongs to the pore-forming toxin family.^42^ *Vc0910* encodes the EIIB/EIIC proteins of the carbohydrate phosphotransferase system (PTS). Previous research has shown that cooperative regulation of PTS is important for bacterial survival and virulence gene expression.^43,44^ Thus, we speculate that MlrA may also contribute to the virulence and/or intestinal survival through regulating hemolysin synthesis and carbon source utilization in *V. cholerae*, and plan to investigate this in future studies.

Unlike most MerR family regulators, a unique binding site for MlrA was identified by us from −234 to −217 bp from the translational start site of *tcpA*. We also showed that, unlike most MerR family regulators, MlrA cannot bind to the – 35 and – 10 elements of the promoter region of the gene (*tcpA*) that it regulates. To date, very few MerR family regulators have been shown to bind to parts of the DNA region other than the – 35 and – 10 elements. For example, MlrA in *E. coli* directly binds to – 146 to – 133 bp segment from the *csgD* translational start site with a palindromic sequence of AAAATTGTACA(12 N)TGTACAATTTT.^45^ In addition, MerR-like protein BldC in *Streptomyces venezuelae* binds to the −50 to −80 bp segment from the translational start site of *whiI*, which plays a critical role in *Streptomyces* differentiation.^35^ Although these two binding sites do not share obvious sequence homology with that of MlrA in *V. cholerae*, all three binding sites contain a conserved AATT motif. It is likely that this AATT motif act as an essential domain for DNA-protein interactions of MerR family regulators.

The C-terminal effector-binding domains of the MerR family transcriptional regulators are highly divergent and can sense a variety of cellular signals.^46^ Several signals sensed by MerR family regulators have been described, including metal ions, oxidative stress, carbonyl and nitrosative stress, and diverse drug-like compounds.^47^ In this study, five potential MerR family regulators were detected in *V. cholerae*. Domain-structure analysis showed that ligand-binding domains were present in the C-terminal effector-binding domain of SoxR, MerR1, ZntR and CueR, but not MlrA. Previous studies have shown that the homolog of ZntR and CueR are metal-sensing transcription regulators that play important roles in the virulence regulation of *Yersinia pseudotuberculosis* and *Pseudomonas aeruginosa*, respectively.^48–50^ The homolog of SoxR is an oxidative stress-sensing transcriptional activator associated with the virulence of *P. aeruginosa* and *Salmonella* Typhimurium.^32,51^ Comparative genomics analysis showed that *zntR* and *cueR* were present in all in *V. cholerae* strains, while *soxR* and *mlrA* were present in all pandemic strains, but absent from some non-pandemic strains (Fig. S3). However, competition assays showed that these regulators had no obvious association with intestinal colonization by *V. cholerae* in infant mice. It is likely that these regulators may contribute to the virulence of *V. cholerae* via mechanisms that cannot be detected in our animal model, or play a role in the environmental survival of bacteria. Unlike SoxR, MerR1, ZntR, and CueR, MlrA does not contain a predicted ligand-binding domain. Furthermore, we showed that *mlrA* expression is activated by Fnr under anaerobic conditions. To the best of our knowledge, this study provides the first demonstration that an anaerobic environment acts as a signal to activate a MerR family regulator.

In conclusion, we describe a novel signaling pathway with detailed mechanisms that links MlrA in *V. cholerae* to anaerobic signals in the small intestine of hosts. These findings provide a paradigm of *V. cholerae* signal perception and virulence regulation in the small intestine, which can be employed to investigate other pathogens in the human gastrointestinal tract.

## Materials and Methods

### Ethics statement

All animal experiments were performed according to the standards set forth by the Guide for the Care and Use of Laboratory Animals. The experimental protocols were approved by the Institutional Animal Care Committee of Nankai University.

### Bacterial strains, plasmids, and growth conditions

Bacterial strains and plasmids used in this study are listed in Table S2 and S3. Briefly *V. cholerae* O1 El Tor strain El2382, isolated in 1994, was provided by Shanghai Municipal Centers for Disease Control and Prevention. *Escherichia coli* BL21-DE3 cells were used as recombinant protein expression hosts. The *E. coli* S17/λpir strain was used for conjugation. The bacterial strains were grown in Luria-Bertani (LB) broth or AKI medium (1.5% Bacto Peptone, 0.4% yeast extract, 0.5% NaCl, and 0.3% NaHCO_3_).^52^ For aerobic condition, bacteria were grown at 37°C with shaking at 180 rpm.^53^ For anaerobic condition, bacteria were grown at 37°C in an anaerobic incubator (YQX-II, Shanghai, China) and oxygen-free nitrogen was used as the carrier gas.^54^ Antibiotics were used as following concentrations: polymyxin B, 40 μg/mL; ampicillin, 50 μg/mL; chloramphenicol, 25 μg/mL.

### Mutant construction and complementation

All primers used in this study are listed in Table S4. Construction of the mutants was performed using the suicide vector pRE112, according to a previously described procedure.^55^ For complementation, genes with native promoters were amplified by PCR and cloned into the pBAD33 vector. For ChIP-qPCR, *mlrA* was amplified along with its promoter regions and cloned into pBAD33 in frame with a C-terminal 3× FLAG-tag. For MlrA purification, *mlrA* was amplified and cloned into the pET28a vector.

### RNA isolation and qRT-PCR

To detect the gene expression *in vivo*, the samples were harvested from the small intestinal tissue of mice. To analyze the expression of virulence genes *in vitro*, the samples were collected from AKI medium under aerobic condition.^52^ To analyze the expression of *mlrA* in response to the anaerobic signal, we collected the samples incubated in LB medium under aerobic or anaerobic condition. When comparing the expression of *mlrA* in WT, Δ*fnr*, and Δ*arcA*, the samples were collected under anaerobic condition. Total RNA was isolated using TRIzol Reagent (15596026; Invitrogen, Waltham, MA, USA), according to the manufacturer’s protocol. Next, total RNA content was determined using a NanoDrop 2000 spectrophotometer (Thermo Fisher Scientific, MA, USA). Three independent experiments were performed. cDNA was synthesized using a PrimeScript™ RT Reagent Kit with gDNA Eraser (Takara, Kusatsu, Japan) according to the manufacturer’s instructions.

The qRT-PCR analysis was conducted with Applied Biosystems ABI 7500 (Applied Biosystems, Waltham, MA, USA) using SYBR green fluorescence dye. In order to normalize samples, the *rrsA* gene was used as a reference control, and relative expression levels were calculated as fold-change values using the 2^−ΔΔCT^ method. Each experiment was performed in triplicate.

### RNA-seq

WT and Δ*mlrA* strains were grown overnight in LB broth and diluted at 1:100 in a fresh AKI medium. After the cultures were grown for 4 h, the bacteria were collected by centrifugation. Total RNA was isolated using TRIzol Reagent (Invitrogen) according to the manufacturer’s protocol. Three independent experiments were performed. Samples were analyzed by Shanghai Majorbio Bio-Pharm Technology Co., Ltd. (Shanghai, China).

### Growth curve

To determine the growth curve of each strain, overnight cultures were diluted to 10^6^ /mL in LB broth in a flask containing 20 mL of LB broth with or without metal ions (1 mM ZnCl_2_, 50 µM CuCl_2_, and 1 µM HgSO_4_).^56,57^ For the aerobic condition, a 200 μL aliquot was added to a 96-well microplate and incubated for 24 hours at 37°C with shaking. The absorbance was recorded at 600 nm. For the anaerobic condition: a 100 μL aliquot was removed from the tube and suitable dilutions were plated on LB agar plates in anaerobic incubator. The absorbance was recorded at 600 nm. The experiments were independently performed three times.

### Electrophoretic mobility shift assay (EMSA)

The 6× His-tagged MlrA protein was expressed and purified in *Escherichia coli* BL21-DE3 cells. Target DNA fragments were amplified and purified using a SPARKeasy Gel DNA Extraction Kit (AE0101 Sparkjade, Jinan, China). Purified DNA fragments (40 ng) were incubated at 30°C for 30 min with 6× His-tagged MlrA protein at concentrations ranging from 0–2 µM, in 20 μL solutions containing binding buffer (10 mM Tris-HCl [pH 7.5], 0.2 mM dithiothreitol, 5 mM MgCl_2_, 10 mM KCl, and 10% glycerol). The protein-DNA fragments were electrophoretically separated on a native polyacrylamide gel at 4°C and 90 V/cm. UV transillumination was used to visualize the protein bands on the gel after 10 minutes of staining in 0.1% GelRed. During effector screening, the purified 6× His-tagged MlrA protein was incubated at 25°C for 10 min with 1 mM ZnCl_2_, 50 µM CuCl_2_, or 1 µM HgSO_4_ before being added to the binding buffer.

### Chromatin immunoprecipitation (ChIP) and ChIP qPCR

Bacteria were grown at 37°C to mid-exponential phase and induced L-arabinose. After centrifugation, 1% formaldehyde was added and incubated at approximately 25°C for 25 min. Then, 0.5 M Glycine was added and mixed. The bacteria were incubated for an additional 5 min to quench the cross-linking reaction. Cross-linked bacteria were harvested and washed three times with ice-cold Tris-buffered saline. The cross-linked bacteria were resuspended in 500 mL lysis buffer (10 mM Tris [pH 7.5], 1 mM EDTA, 100 mM NaCl, 1 mM protease inhibitor cocktail, 1 mg/mL lysozyme, 0.1 mg/ml RNase A) and incubated at 37°C for 30 min. Then, immunoprecipitation (IP) buffer (100 mM Tris-HCl [pH 7.5], 200 mM NaCl, 1 mM EDTA, 2% v/v Triton X-100, 1 mM phenylmethane sulfonyl fluoride) was incubated, and the lysates were further sonicated to generate DNA fragments of approximately 300 bp length. After centrifugation at 12,000 g for 10 minutes, supernatants were incubated with anti-3× FLAG antibody. (#F1804; Sigma-Aldrich, St. Louis, MO, USA) and protein A magnetic beads (Invitrogen; #10002D). DNA samples were subsequently purified using a PCR purification kit (#28104; Qiagen, Hilden, Germany). In order to determine MlrA-binding peak enrichment, a ChIP-qPCR was carried out using an ABI 7500 sequence-detection system. The *rrsA* gene (nonspecific enrichment) was used as a reference. The relative enrichment of candidate targets was calculated as fold-enrichment using the formula 2^−ΔΔCT^. The results were reported as the average enrichment of three biological replicates.

### Dye primer-based DNase I footprinting assay

DNase I footprinting procedures were modified from published procedures. Approximately 200-bp fragments of the *tcpA* promoter regions were generated by PCR with 6-FAM primers. Various amounts of MlrA protein were added to 40 nanograms of 6-FAM-labeled tcpA promoter in a binding buffer (10 mM Tris-HCl [pH 7.5], 0.2 mM dithiothreitol, 5 mM MgCl_2_, 10 mM KCl, and 10% glycerol) from 0 to 1 mM. 0.05 U DNase I (Sigma; AMPD1) was added to a 20-μL solution for 10 min at 37°C. The reaction was stopped by heating at 85°C for 10 min in the presence of 250 mM ethylenediaminetetraacetic acid (EDTA). DNA fragments were purified using the QIAquick PCR Purification Kit (Qiagen; #28104) and eluted in 15 μL distilled water. The samples were analyzed by MAP Biotech Co., Ltd. (Shanghai, China). The results were analyzed using a peak scanner (Applied Biosystems).

### Western blotting

The *V. cholerae* strains were grown overnight and diluted 1:100 in a fresh AKI medium. After being grown anaerobically for 4 h and reaching an optical density of 0.2, the cultures were shaken for 2–2.5 h to reach an optical density of 1.0. Bacterial cells were harvested, washed, and resuspended in phosphate-buffered saline (PBS) at 4°C, sonicated for 15 cycles of 30s on/off at 95% power. The cell debris was removed by centrifugation at 12,000 × g for 10 min at 4°C. To quantify the supernatants, we used BSA method and separated equal amounts of the total protein using SDS-PAGE with a 4–12% gel and transferred onto PVDF membranes (Bio-Rad) by electroblotting. Blots for RNA polymerase (RNAP), cholera toxin, and TcpA were incubated with anti-RNA polymerase beta (ab191598), anti-cholera toxin (ab123129), and anti-TcpA monoclonal antibodies (Willget Biotech Co., Ltd., Shanghai, China), respectively, at a dilution of 1:2000. To detect proteins, the blots were incubated with goat anti-rabbit IgG secondary antibodies (1:5000 dilution; Sparkjade, EF0002) with horseradish peroxidase. Detection was performed using a Sparkjade ECL Plus (ED0016; Sparkjade) detection system. Images were acquired using an Amersham Imager 600 system (General Electric).

### Intestinal colonization assay

Five-day-old CD-1 mice were purchased from Beijing Vital River Laboratory Animal Technology (Beijing, China) and placed in incubators at 30°C. An *in vivo* competition assay for intestinal colonization was performed as previously described with minor modification.^29^ Briefly, the *V. cholerae lacZ*+ strains (wild-type and mutants) and *lacZ*− strains (Δ*lacZ*) were grown overnight at 37°C with aeration in LB broth. Approximately 10^5^ *lacZ*+ strains were mixed with an equal number of *lacZ*− cells and the mixtures were intragastrically administered to groups of eight anesthetized mice. In order to enumerate the recovered bacteria and obtain output ratios, the small intestine was removed, weighed, homogenized, and plated on LB agar plates containing 5-bromo-4-chloro-3-indoyl-β-D-galactopyranoside (X-gal). The competitive index (CI) was determined as the output ratio of *lacZ*+ to *lacZ*− cells, divided by the input ratio of *lacZ*+ to *lacZ*− cells.

### Statistical analyses

Data were analyzed using a t-test, two-way ANOVA or Mann–Whitney U test, and differences were evaluated using independent samples t-tests. Values of *p* < .05, 0.01, or 0.001 were considered statistically significant (*), highly significant (**), or extremely significant (***), respectively, and n.s. represents no significance. Figures were drawn using Origin 8.5 (Origin Lab Corporation).

## Supplementary Material

Supplemental Material

## Data Availability

RNA sequencing data generated in this study are available in the NCBI SRA database. Accession to cite SRA data: PRJNA870313 https://www.ncbi.nlm.nih.gov/bioproject/PRJNA870313.

## References

[1] Clemens JD, Nair GB, Ahmed T, Qadri F, Holmgren J. Cholera. Lancet. 2017;390:1539–14. PMID: 28302312. doi:10.1016/S0140-6736(17)30559-7. 28302312

[2] Mogasale V, Mogasale VV, Hsiao A. Economic burden of cholera in Asia. Vaccine. 2020;38(Suppl1):A160–A166. doi:10.1016/j.vaccine.2019.09.099. PMID: 31611097 31611097

[3] Mutreja A, Dougan G. Molecular epidemiology and intercontinental spread of cholera. Vaccine. 2020;38(Suppl 1):A46–A51. doi:10.1016/j.vaccine.2019.07.038. PMID: 31345641 31345641

[4] Conner JG, Teschler JK, Jones CJ, Yildiz FH. Staying alive: *vibrio cholerae*’s cycle of environmental survival, transmission, and dissemination. Microbiol Spectr. 2016;4(2). doi:10.1128/microbiolspec.VMBF-0015-2015. PMID: 27227302 PMC4888910 27227302

[5] Alam M, Sultana M, Nair GB, Sack RB, Sack DA, Siddique AK, Ali A, Huq A, Colwell RR. Toxigenic *Vibrio cholerae* in the aquatic environment of Mathbaria, Bangladesh. Appl Environ Microbiol. 2006;72(4):2849–2855. doi:10.1128/AEM.72.4.2849-2855.2006. PMID: 16597991 16597991 PMC1449004

[6] Cho JY, Liu R, Macbeth JC, Hsiao A. The interface of and the gut microbiome. Gut Microbes. 2021;13(1):1937015. doi:10.1080/19490976.2021.1937015. PMID: 34180341 34180341 PMC8244777

[7] Waldor MK, Mekalanos JJ. Lysogenic conversion by a filamentous phage encoding cholera toxin. Science. 1996;272(5270):1910–1914. doi:10.1126/science.272.5270.1910. PMID: 8658163 8658163

[8] Yu RR, DiRita VJ. Regulation of gene expression in *Vibrio cholerae* by ToxT involves both antirepression and RNA polymerase stimulation. Mol Microbiol. 2002;43(1):119–134. doi:10.1046/j.1365-2958.2002.02721.x. PMID: 11849541 11849541

[9] Ghasemi M, Bakhshi B, Khashei R, Soudi S, Boustanshenas M. *Vibrio cholerae* toxin coregulated pilus provokes inflammatory responses in Coculture model of Caco-2 and peripheral blood mononuclear cells (PBMC) leading to increased colonization. Microbiol Immunol. 2021;65(6):238–244. doi:10.1111/1348-0421.12889. PMID: 33913531 33913531

[10] Herrington DA, Hall RH, Losonsky G, Mekalanos JJ, Taylor RK, Levine MM. Toxin, toxin-coregulated pili, and the *toxR* regulon are essential for *Vibrio cholerae* pathogenesis in humans. J Exp Med. 1988;168(4):1487–1492. doi:10.1084/jem.168.4.1487. PMID: 2902187 2902187 PMC2189073

[11] Kirn TJ, Lafferty MJ, Sandoe CM, Taylor RK. Delineation of pilin domains required for bacterial association into microcolonies and intestinal colonization by *Vibrio cholerae*. Mol. Microbiol. 2000;35:896–910. PMID: 10692166. doi:10.1046/j.1365-2958.2000.01764.x. 10692166

[12] Manning PA. The *tcp* gene cluster of *Vibrio cholerae*. Gene. 1997;192(1):63–70. doi:10.1016/s0378-1119(97)00036-x. PMID: 9224875 9224875

[13] Martin RG, Rosner JL. The AraC transcriptional activators. Curr Opin Microbiol. 2001;4(2):132–137. doi:10.1016/s1369-5274(00)00178-8. PMID: 11282467 11282467

[14] Calkins AL, Demey LM, Karslake JD, Donarski ED, Biteen JS, DiRita VJ. Independent promoter recognition by TcpP precedes cooperative promoter activation by TcpP and ToxR. MBio. 2021;12(5):e0221321. doi:10.1128/mBio.02213-21. Epub 2021 Sep 7. PMID: 34488449 34488449 PMC8546550

[15] Mey AR, Wyckoff EE, Kanukurthy V, Fisher CR, Payne SM. Iron and Fur regulation in *Vibrio cholerae* and the role of Fur in virulence. Infect Immun. 2005;73(12):8167–8178. doi:10.1128/IAI.73.12.8167-8178.2005. PMID: 16299312 16299312 PMC1307094

[16] Gao H, Zhang J, Lou J, Li J, Qin Q, Shi Q, Zhang Y, Kan B. Direct binding and regulation by Fur and HapR of the intermediate regulator and virulence factor genes within the ToxR virulence regulon in *Vibrio cholerae*. Front Microbiol. 2020;11:709. PMID: 32362889. doi:10.3389/fmicb.2020.00709. 32362889 PMC7181404

[17] Xu X, Stern AM, Liu Z, Kan B, Zhu J. Virulence regulator AphB enhances *toxR* transcription in *Vibrio cholerae*. BMC Microbiol. 2010;10:3. PMID: 20053280. doi:10.1186/1471-2180-10-3. 20053280 PMC2806343

[18] Acosta N, Pukatzki S, Raivio TL. The Cpx system regulates virulence gene expression in *Vibrio cholerae*. Infect Immun. 2015;83(6):2396–2408. doi:10.1128/IAI.03056-14. PMID: 25824837 25824837 PMC4432731

[19] Liu Y, Xu T, Wang Q, Huang J, Zhu Y, Liu X, Liu R, Yang B, Zhou K. *Vibrio cholerae* senses human enteric α-defensin 5 through a CarSR two-component system to promote bacterial pathogenicity. Commun Biol. 2022;5(1):559. doi:10.1038/s42003-022-03525-3. PMID: 35676416 35676416 PMC9178039

[20] Hsiao A, Zhu J. Pathogenicity and virulence regulation of *Vibrio cholerae* at the interface of host-gut microbiome interactions. Virulence. 2020;11(1):1582–1599. doi:10.1080/21505594.2020.1845039. PMID: 33172314 33172314 PMC7671094

[21] Crompton DW, Shrimpton DH, Silver IA. Measurements of the oxygen tension in the lumen of the small intestine of the domestic duck. J Exp Biol. 1965;43(3):473–478. doi:10.1242/jeb.43.3.473. PMID: 5893422 5893422

[22] Byndloss MX, Olsan EE, Rivera-Chávez F, Tiffany CR, Cevallos SA, Lokken KL, Torres TP, Byndloss AJ, Faber F, Gao Y, et al. Microbiota-activated PPAR-γ signaling inhibits dysbiotic Enterobacteriaceae expansion. Science. 2017;357(6351):570–575. PMID: 28798125. doi:10.1126/science.aam9949. 28798125 PMC5642957

[23] Wallace N, Zani A, Abrams E, Sun Y. The Impact of oxygen on bacterial enteric pathogens. Adv Appl Microbiol. 2016;95:179–204. PMID: 27261784. doi:10.1016/bs.aambs.2016.04.002. 27261784

[24] Marrero K, Sánchez A, Rodríguez-Ulloa A, González LJ, Castellanos-Serra L, Paz-Lago D, Campos J, Rodríguez BL, Suzarte E, Ledón T, et al. Anaerobic growth promotes synthesis of colonization factors encoded at the Vibrio pathogenicity island in *Vibrio cholerae* El Tor. Res Microbiol. 2009;160(1):48–56. PMID: 19015025. doi:10.1016/j.resmic.2008.10.005. 19015025

[25] Lee KM, Park Y, Bari W, Yoon MY, Go J, Kim SC, Lee HI, Yoon SS. Activation of cholera toxin production by anaerobic respiration of trimethylamine N-oxide in *Vibrio cholerae*. J Biol Chem. 2012;287(47):39742–39752. doi:10.1074/jbc.M112.394932. PMID: 23019319 23019319 PMC3501055

[26] Baksh KA, Zamble DB. Allosteric control of metal-responsive transcriptional regulators in bacteria. J Biol Chem. 2020;295(6):1673–1684. doi:10.1074/jbc.REV119.011444. PMID: 31857375 31857375 PMC7008368

[27] Brown NL, Stoyanov JV, Kidd SP, Hobman JL. The MerR family of transcriptional regulators. FEMS Microbiol Rev. 2003;27(2–3):145–163. doi:10.1016/S0168-6445(03)00051-2. PMID: 12829265 12829265

[28] Lee PE, Demple B, Barton JK. DNA-mediated redox signaling for transcriptional activation of SoxR. Proc Natl Acad Sci U S A. 2009;106(32):13164–13168. doi:10.1073/pnas.0906429106. PMID: 19651620 19651620 PMC2726364

[29] Sheehan LM, Budnick JA, Roop RM, Caswell CC. Coordinated zinc homeostasis is essential for the wild-type virulence of *Brucella abortus*. J Bacteriol. 2015;197(9):1582–1591. doi:10.1128/JB.02543-14. PMID: 25691532 25691532 PMC4403653

[30] Jiang X, Li X, Sun S, Jiang L. The transcriptional regulator VarN contributes to *Salmonella* Typhimurium growth in macrophages and virulence in mice. Res Microbiol. 2018;169(4–5):214–221. doi:10.1016/j.resmic.2018.03.003. PMID: 29751061 29751061

[31] Han Y, Wang T, Chen G, Pu Q, Liu Q, Zhang Y, Xu L, Wu M, Liang H. A *Pseudomonas aeruginosa* type VI secretion system regulated by CueR facilitates copper acquisition. PLoS Pathog. 2019;15(12):e1008198. doi:10.1371/journal.ppat.1008198. PMID: 31790504 31790504 PMC6907878

[32] Palma M, Zurita J, Ferreras JA, Worgall S, Larone DH, Shi L, Campagne F, Quadri LE. *Pseudomonas aeruginosa* SoxR does not conform to the archetypal paradigm for SoxR-dependent regulation of the bacterial oxidative stress adaptive response. Infect Immun. 2005;73(5):2958–2966. doi:10.1128/IAI.73.5.2958-2966.2005. PMID: 15845502 15845502 PMC1087365

[33] Hu D, Yin Z, Yuan C, Yang P, Qian C, Wei Y, Zhang S, Wang Y, Yuan J, Wang M, et al. Changing molecular epidemiology of *Vibrio cholerae* outbreaks in Shanghai, China. MSystems. 2019;4(6):e00561–19. PMID: 31771974. doi:10.1128/mSystems.00561-19. 31771974 PMC6880041

[34] Fu Y, Waldor MK, Mekalanos JJ. Tn-Seq analysis of *Vibrio cholerae* intestinal colonization reveals a role for T6SS-mediated antibacterial activity in the host. Cell Host Microbe. 2013;14(6):652–663. doi:10.1016/j.chom.2013.11.001. PMID: 24331463 24331463 PMC3951154

[35] Schumacher MA, den Hengst CD, Bush MJ, Le TBK, Tran NT, Chandra G, Zeng W, Travis B, Brennan RG, Buttner MJ. The MerR-like protein BldC binds DNA direct repeats as cooperative multimers to regulate Streptomyces development. Nat Commun. 2018;9(1):1139. doi:10.1038/s41467-018-03576-3. PMID: 29556010 29556010 PMC5859096

[36] Hobman JL. MerR family transcription activators: similar designs, different specificities. Mol Microbiol. 2007;63(5):1275–1278. doi:10.1111/j.1365-2958.2007.05608.x. PMID: 17302809 17302809

[37] Rolfs A, Hediger MA. Intestinal metal ion absorption: an update. Curr Opin Gastroen. 2001;17(2):177–183. doi:10.1097/00001574-200103000-00014. PMID: 11224676 11224676

[38] Kovacikova G, Lin W, Skorupski K. The LysR-type virulence activator AphB regulates the expression of genes in *Vibrio cholerae* in response to low pH and anaerobiosis. J Bacteriol. 2010;192(16):4181–4191. doi:10.1128/JB.00193-10. Epub 2010 Jun 18. PMID: 20562308 20562308 PMC2916415

[39] Yang Y, Liu C, Zhou W, Shi W, Chen M, Zhang B, Schatz DG, Hu Y, Liu B. Structural visualization of transcription activated by a multidrug-sensing MerR family regulator. Nat Commun. 2021;12(1):2702. doi:10.1038/s41467-021-22990-8. PMID: 33976201 33976201 PMC8113463

[40] Mika F, Hengge R. Small RNAs in the control of RpoS, CsgD, and biofilm architecture of *Escherichia coli*. RNA Biol. 2014;11(5):494–507. doi:10.4161/rna.28867. Epub 2014 Apr 25. PMID: 25028968 25028968 PMC4152358

[41] Rivera-Chávez F, Mekalanos JJ. Cholera toxin promotes pathogen acquisition of host-derived nutrients. Nature. 2019;572(7768):244–248. doi:10.1038/s41586-019-1453-3. PMID: 31367037 31367037 PMC6727848

[42] Wang G, Fan C, Wang H, Jia C, Li X, Yang J, Zhang T, Gao S, Min X, Huang J. Type VI secretion system-associated FHA domain protein TagH regulates the hemolytic activity and virulence of *Vibrio cholerae*. Gut Microbes. 2022;14(1):2055440. doi:10.1080/19490976.2022.2055440. PMID: 35383540 35383540 PMC8993066

[43] Lee HY, Yoon CK, Cho YJ, Lee JW, Lee KA, Lee WJ, Seok YJ. A mannose-sensing AraC-type transcriptional activator regulates cell-cell aggregation of V*ibrio cholerae*. NPJ Biofilms Microbi. 2022;8(1):65. doi:10.1038/s41522-022-00331-x. PMID: 35987769 PMC9392796 35987769

[44] Wang Q, Millet YA, Chao MC, Sasabe J, Davis BM, Waldor MK. A genome-wide screen reveals that the *Vibrio cholerae* phosphoenolpyruvate phosphotransferase system modulates virulence gene expression. Infect Immun. 2015;83(9):3381–3395. doi:10.1128/IAI.00411-15. PMID: 26056384 26056384 PMC4534656

[45] Ogasawara H, Yamamoto K, Ishihama A. Regulatory role of MlrA in transcription activation of *csgD*, the master regulator of biofilm formation in *Escherichia coli*. FEMS Microbiol Lett. 2010;312(2):160–168. doi:10.1111/j.1574-6968.2010.02112.x. PMID: 20874755 20874755

[46] Liu X, Hu Q, Yang J, Huang S, Wei T, Chen W, He Y, Wang D, Liu Z, Wang K, et al. Selective cadmium regulation mediated by a cooperative binding mechanism in CadR. Proc Natl Acad Sci U S A. 2019;116:20398–20403. PMID: 31548408. doi:10.1073/pnas.1908610116. 31548408 PMC6789929

[47] Pomposiello PJ, Demple B. Redox-operated genetic switches: the SoxR and OxyR transcription factors. Trends Biotechnol. 2001;19(3):109–114. doi:10.1016/s0167-7799(00)01542-0. PMID: 11179804 11179804

[48] Hobman JL, Wilkie J, Brown NL. A design for life: prokaryotic metal-binding MerR family regulators. Biometals. 2005;18(4):429–436. doi:10.1007/s10534-005-3717-7. PMID: 16158235 16158235

[49] Wang T, Chen K, Gao F, Kang Y, Chaudhry MT, Wang Z, Wang Y, Shen X. ZntR positively regulates T6SS4 expression in *Yersinia pseudotuberculosis*. J Microbiol. 2017;55:448–456. PMID: 28281200. doi:10.1007/s12275-017-6540-2. 28281200

[50] Bagchi A. Structural characterizations of metal ion binding transcriptional regulator CueR from opportunistic pathogen *Pseudomonas aeruginosa* to identify its possible involvements in virulence. Appl Biochem Biotechnol. 2015;175(2):649–656. doi:10.1007/s12010-014-1304-5. PMID: 25342258 25342258

[51] Wang P, Zhang H, Liu Y, Lv R, Liu X, Song X, Wang J, Jiang L. SoxS is a positive regulator of key pathogenesis genes and promotes intracellular replication and virulence of *Salmonella* Typhimurium. Microb Pathog. 2020;139:103925. PMID: 31838175. doi:10.1016/j.micpath.2019.103925. 31838175

[52] Iwanaga M, Yamamoto K, Higa N, Ichinose Y, Nakasone N, Tanabe M. Culture conditions for stimulating cholera toxin production by *Vibrio cholerae* O1 El Tor. Microbiol Immunol. 1986;30(11):1075–1083. doi:10.1111/j.1348-0421.1986.tb03037.x. PMID: 3543624 3543624

[53] Liu Y, Liu B, Xu T, Wang Q, Li W, Wu J, Zheng X, Liu B, Liu R, Liu X, et al. A fructose/H^+^ symporter controlled by a LacI-type regulator promotes survival of pandemic *Vibrio cholerae* in seawater. Nat Commun. 2021;12(1):4649. PMID: 34330925. doi:10.1038/s41467-021-24971-3. 34330925 PMC8324912

[54] Wagner AO, Markt R, Mutschlechner M, Lackner N, Prem EM, Praeg N, Illmer P. Medium preparation for the cultivation of microorganisms under strictly anaerobic/anoxic conditions. Jove-J Vis Exp. 2019;150 PMID: 31475968. doi:10.3791/60155. PMC6796894 31475968

[55] Xu T, Cao H, Zhu W, Wang M, Du Y, Yin Z, Chen M, Liu Y, Yang B, Liu B. RNA-seq-based monitoring of gene expression changes of viable but non-culturable state of *Vibrio cholerae* induced by cold seawater. Env Microbiol Rep. 2018;10(5):594–604. doi:10.1111/1758-2229.12685. PMID: 30058121 30058121

[56] Stoyanov JV, Hobman JL, Brown NL. CueR (YbbI) of *Escherichia coli* is a MerR family regulator controlling expression of the copper exporter CopA. Mol Microbiol. 2001;39(2):502–511. doi:10.1046/j.1365-2958.2001.02264.x. PMID: 11136469 11136469

[57] Yang M, Liu Z, Hughes C, Stern AM, Wang H, Zhong Z, Kan B, Fenical W, Zhu J. Bile salt-induced intermolecular disulfide bond formation activates *Vibrio cholerae* virulence. Proc Natl Acad Sci U S A. 2013;110(6):2348–2353. doi:10.1073/pnas.1218039110. PMID: 23341592 23341592 PMC3568309

