# Supplementary material for: MlrA, a MerR family regulator in Vibrio cholerae, senses the anaerobic signal in the small intestine of the host to promote bacterial intestinal colonization
Source: Gut Microbes. 2022 Nov 11;14(1):2143216. doi: 10.1080/19490976.2022.2143216 (PMC9662190; doi:10.1080/19490976.2022.2143216)
Supplement: Supplemental Material [file KGMI_A_2143216_SM2206.zip › Supplemental meterial.docx]

**Supplemental Meterials for**

**MlrA, a** [**MerR family regulator in *Vibrio cholerae*, senses the anaerobic signal in the small intestine of the host to promote bacterial intestinal colonization**](https://pubmed.ncbi.nlm.nih.gov/22730129/)

Jialin Wu^a,b,†^, Yutao Liu^a,b,c,†^, Wendi Li^a,b^, Fan Li^a,b^, Ruiying Liu^a,b^, Hao Sun^a,b^, Jingliang Qin^a,b^, Xiaohui Feng^a,b^, Di Huang ^a,b,c,*^, Bin Liu^a,b,c,*^

*^a^TEDA Institute of Biological Sciences and Biotechnology, Nankai University, Tianjin, China*

*^b^Key Laboratory of Molecular Microbiology and Technology, Nankai University, Ministry of Education, Tianjin, China*

*^c^Nankai International Advanced Research Institute, Nankai University Shenzhen, China.*

^†^ Jialin Wu and Yutao Liu contributed equally to this work.

*Corresponding author. Email: liubin1981@nankai.edu.cn (Bin Liu); huangdi@nankai.edu.cn (Di Huang).

**Supplemental Figures**


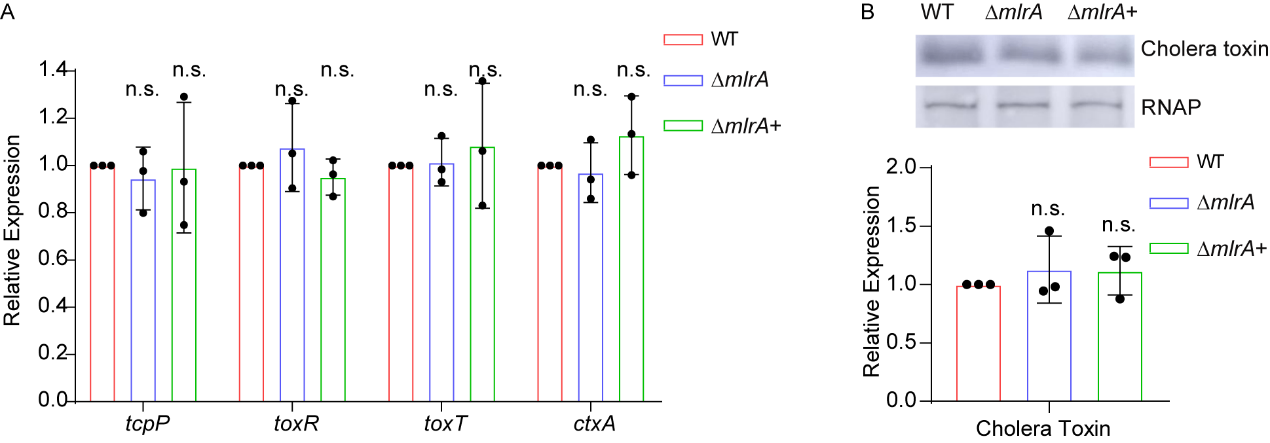


**Supplemental Figure 1. MlrA has no influence on** **cholera toxin.**

(A) qRT-PCR expression level of virulence genes (*tcpP*, *toxR*, *toxT* and *ctxA*) in WT, Δ*mlrA* and Δ*mlrA*+ at the logarithmic phase in AKI media. Data represent the mean ± SD (n = 3). (B) Representative western blotting image and quantitative analysis of cholera toxin in WT, Δ*mlrA* and Δ*mlrA*+ in AKI medium. RNA polymerase (RNAP) was used as a loading control. Data represent the mean ± SD (n = 3). Two-way ANOVA was used to calculate *P* values. * *P* < 0.05, ** *P* < 0.01, *** *P* < 0.001; ns, not significant.


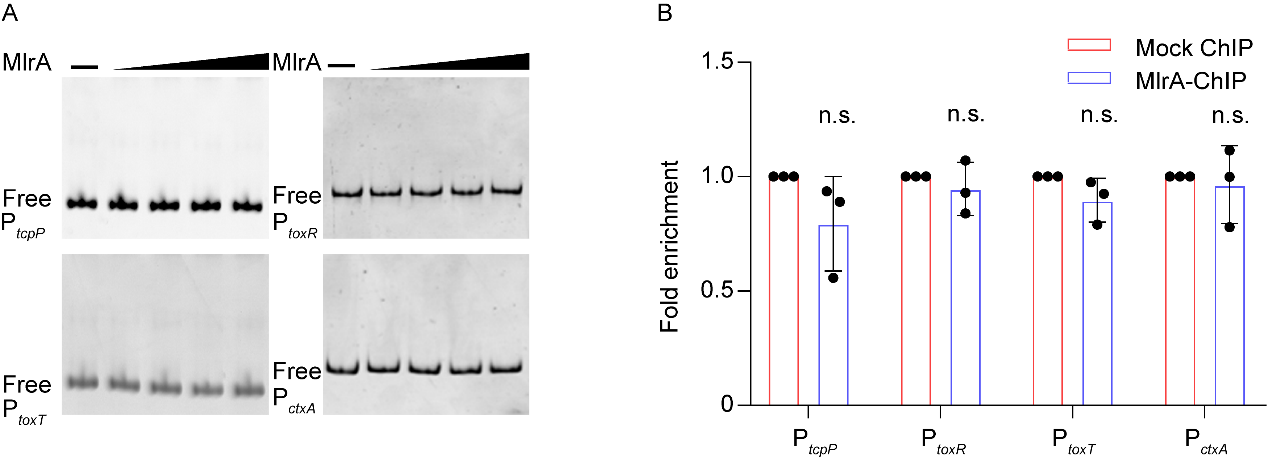


**Supplemental Figure 2. MlrA directly promotes the expression of *tcpA*.**

(A) EMSA of the specific binding of purified MlrA protein to the promoter region of *tcpP, toxR, toxT* and *ctxA*. (B) The fold enrichment of the promoters for *tcpP, toxR, toxT* and *ctxA* in the chromatin immunoprecipitation assay. Significance is determined by two-tailed unpaired Student’s t test. * *P* < 0.05, ** *P* < 0.01, *** *P* < 0.001; ns, not significant.


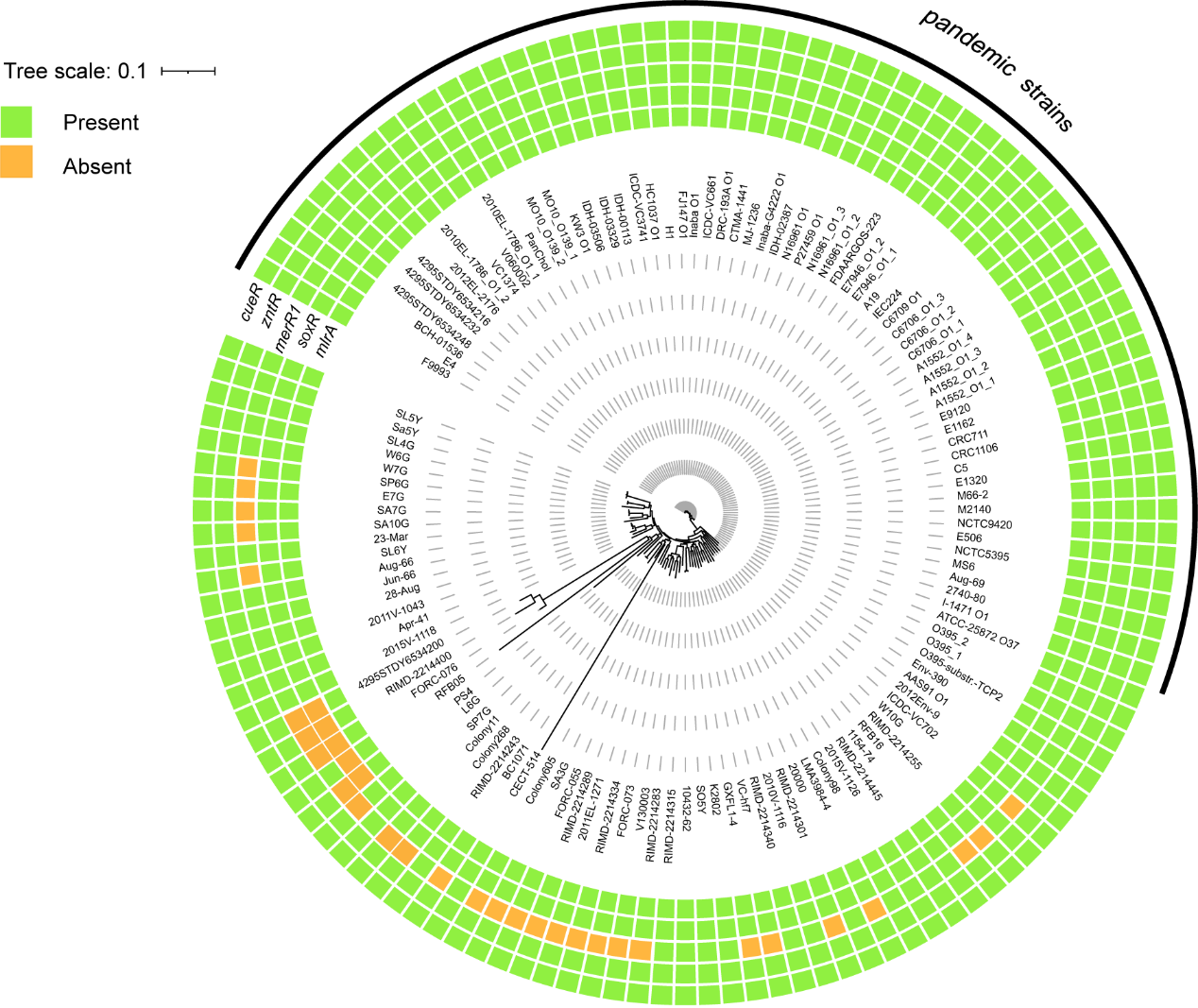


**Supplemental Figure 3. The analysis on the distribution of *mlrA*, *soxR*, *merR1*, *zntR* and *cueR* in 127 representative *V. cholerae* strains.** Circles represent the *mlrA*, *soxR*, *merR1*, *zntR* and *cueR* from inner to outer order. In the corresponding strains, the presence of the gene is indicated in green, and the absence of the gene is indicated in orange. The area surrounded by the black curves represents the pandemic strains.

**Supplemental table 1. Strains used in this study.**

| **Strains** | **Genotype or description** | **Source** |
| --- | --- | --- |
| E12382 | *Vibrio Cholerae* O1 El Tor strain E12382 | Shanghai CDC* |
| S-17 | *E. coli* S17-1/λpir strain | Lab collection |
| BL21 | Expression strain | Lab collection |
| DH5α | *E. coli* DH5α/λpir strain | Lab collection |
| Δ*mlrA* | *mlrA* deletion mutant in E12382 | This work |
| Δ*zntR* | *zntR* deletion mutant in E12383 | This work |
| Δ*soxR* | *soxR* deletion mutant in E12384 | This work |
| Δ*cueR* | *cueR* deletion mutant in E12385 | This work |
| Δ*merR*1 | *merR1* deletion mutant in E12386 | This work |
| Δ*mlrA*+ | Δ*mlrA* containing pBAD33-*mlrA* | This work |
| Δ*arcA* | *arcA* deletion mutant in E12382 | This work |
| Δ*fnr* | *fnr* deletion mutant in E12387 | This work |
| BL21-*mlrA* | BL21(DE3) containing pET28a- *mlrA* | This work |

* , Shanghai CDC, Shanghai Municipal Center for Disease Control & Prevention, China.

**Supplemental table 2. plasmids used in this study.**

| Name | Desciption | Source/Reference |
| --- | --- | --- |
| pBAD33 | Bacteria expression vector, Cm^R^ | Lab collection |
| pRE112 | Suicide vector for mutation, Cm^R^ | Lab collection |
| pET28a | T7 expression vector, Km^R^ | Lab collection |
| pBAD33-*mlrA* | pBAD33 carrying *mlrA* from E12382，Cm^R^ | This work |
| pET28a-*mlrA* | pET28a carrying *mlrA* from E12382，Km^R^ | This work |

**Supplemental table 3. Primers used in this study (5'-3').**

| Primers for gene mutation | | |
| --- | --- | --- |
| *mlrA* | P1 | TCC CCCGGG AGATGGTCGATTGAATGATCCGGT |
| *mlrA* | P2 | CCAAACCAGTCTCATACCAATTCCAACGTAGTTTCAACTCCTGAGGAT |
| *mlrA* | P3 | ATCCTCAGGAGTTGAAACTACGTTGGAATTGGTATGAGACTGGTTTGG |
| *mlrA* | P4 | GC TCTAGA GGCGGCTGAAATAGAGTGAGCCAT |
| *zntR* | P1 | GCTCTAGAGCTGCAGGGTTTTGACGCGTCC |
| *zntR* | P2 | GAATATAAATTAGGCGTTATTTCTTCCTCCTCCTCCCTGACTACT |
| *zntR* | P3 | AGTAGTCAGGGAGGAGGAGGAAGAAATAACGCCTAATTTATATTC |
| *zntR* | P4 | CTAGTCTAGA TCACCACATAGCTGAGGTTTTCTT |
| *soxR* | P1 | GCTCTAGAGCGATTACGGCTCGACCCTTATTATTCC |
| *soxR* | P2 | TTCGGTCGATGGGAGACAAATCATACTAGATTTAACACTGTACTTAG |
| *soxR* | P3 | CTAAGTACAGTGTTAAATCTAGTATGATTTGTCTCCCATCGACCGAA |
| *soxR* | P4 | CTAGTCTAGA GCTGAGCGGCTGCTAATAATCCCA |
| *cueR* | P1 | CGGGGTACCGTTGCTGTTCAAGCGCAACCAACC |
| *cueR* | P2 | GCAACTTCAAGTCGGAACTAAGATCGACACATAAAAAATCACAGAG |
| *cueR* | P3 | CTCTGTGATTTTTTATGTGTCGATCTTAGTTCCGACTTGAAGTTGC |
| *cueR* | P4 | CTAGTCTAGA GTGCCCCGTGGTAGAGCTACTC |
| *merR1* | P1 | CGGGGTACCTCGGGGTGATCGCTTTCCCTGTTAA |
| *merR1* | P2 | TGCCTTTTGAGCTCAGATAAAGCAGACTTAAGAATGAATTTTTCTTTA |
| *merR1* | P3 | TAAAGAAAAATTCATTCTTAAGTCTGCTTTATCTGAGCTCAAAAGGCA |
| *merR1* | P4 | CTAGTCTAGAGCGCCATTACCTTCTGCCACAAGT |
| *fnr* | P1 | TCCCCCGGGATTGTATTAGGCCTTTACATCGGA |
| *fnr* | P2 | TCATTAAGAGCTACATCATTGAAGAACAACCTTTCACTAATTGATGTA |
| *fnr* | P3 | TACATCAATTAGTGAAAGGTTGTTCTTCAATGATGTAGCTCTTAATGA |
| *fnr* | P4 | GGGGTACCAGCATGTCGTGTTTGCGAGTCGCT |
| *arcA* | P1 | TCCCCCGGGCCAATCATAATCGATCAAGCATTG |
| *arcA* | P2 | TCTAATTAGATATAAAAGAAGAGGTAGCGTAACCTAAACTTGTGAAA |
| *arcA* | P3 | TTTCACAAGTTTAGGTAACGCTACCTCTTCTTTTATATCTAATTAGA |
| *arcA* | P4 | GCTCTAGAGAAGATGACGCAACATCATGCCGG |
| Primers for pBAD33 identifying | | |
| pBAD33 | F | ATGCCATAGCATTTTTATCC |
| pBAD33 | R | GATTTAATCTGTATCAGG |
| pet28a | F | TAATACGACTCACTATAGGG |
| pet28a | R | GCTAGTTATTGCTCAGCGG |
| pRE112 | F | CACTGTTCGTCCATTTCCG |
| pRE112 | R | TTCGTCTCAGCCAATCCCT |
| Primers for complement construction | | |
| *mlrA* | F | CGAGCTCAGTATCCACTGGATTCTTTTATCC |
| *mlrA* | R | TCCCCCGGGCTAAGATTGTAGCTGTTGTAGCAC |
| Primers for protein purification | | |
| *mlrA* | F | GGAATTCCATATGATGGTTTGTGATGAAAAACGTTAC |
| *mlrA* | R | CCGCTCGAGCTAAGATTGTAGCTGTTGTAGCAC |
| Primers for mutant verification | | |
| *mlrA* | F | TTTGCAAGGTTATTTCAAGCGC |
| *mlrA* | R | ATGAGACTGGTTTGGTTTCGTC |
| *fnr* | F | GATGCCGTAAAGCTGATGATTAAT |
| *fnr* | R | CATCATGGTTGATATCAGCCACGA |
| *arcA* | F | TCCCCCGGGCCAATCATAATCGATCAAGCATTG |
| *arcA* | R | GCTCTAGAGAAGATGACGCAACATCATGCCGG |
| *zntR* | F | TGCAGGGTTTTGACGCGTCC |
| *zntR* | R | TCACCACATAGCTGAGGTTTTCTT |
| *soxR* | F | GATTACGGCTCGACCCTTATTATTCC |
| *soxR* | R | GCTGAGCGGCTGCTAATAATCCCA |
| *cueR* | F | GTTGCTGTTCAAGCGCAACCAACC |
| *cueR* | R | GTGCCCCGTGGTAGAGCTACTC |
| *merR1* | F | TCGGGGTGATCGCTTTCCCTGTTAA |
| *merR1* | R | GCGCCATTACCTTCTGCCACAAGT |
| Primers for qRT-PCR | | |
| 16S | F | ACCTTACCTACTCTTGACATCCA |
| 16S | R | CCCAACATTTCACAACACGAG |
| *mlrA* | F | CGATTGGGAAAGTGGGCGCGTT |
| *mlrA* | R | CGCTCAAGGGCTTCTGTGACTGGT |
| *fnr* | F | ATGCCGAAACTGCGTCAACAAA |
| *fnr* | R | CTCTTGGGCTAAAACCGCGTTG |
| *tcpP* | F | ATGGGGTATGTCCGCGTGAT |
| *tcpP* | R | TTTGGACAGGGGGCAGGATG |
| *toxR* | F | GCTCAAGCCGATAGAAGT |
| *toxR* | R | GATGCGTAAGGTTATGTTTT |
| *toxT* | F | CGTTGGGCAGATATTTGTGGTG |
| *toxT* | R | CACTTGGTGCTACATTCATGGTTG |
| *ctxA* | F | CTCAGACGGGATTTGTTAGGC |
| *ctxA* | R | CTATCTCTGTAGCCCCTATTACG |
| *tcpA* | F | GGTGTGGTCTCAGCGGGTGTTG |
| *tcpA* | R | AGGATTCTTTGCCTCATCAGCTGA |
| *tcpF* | F | TGACGCATACCCATCGACAGAA |
| *tcpF* | R | CCCTTGTCGGTATTTCCCAATCGA |
| EMSA-promoter | | |
| *kana* | F | CATACGCTTGATCCGGCTAC |
| *kana* | R | CGGCCATTTTCCACCATGATAT |
| *P_mlrA_* | F | CCACTGGATTCTTTTATCCAAGAG |
| *P_mlrA_* | R | AACGTAGTTTCAACTCCTGAGGAT |
| *P_tcpP_* | F | AACCGAATGAATTATAATGAG |
| *P_tcpP_* | R | TCATTGGATCTTGTGCATAATA |
| *P_toxR_* | F | CAATTGATCCATTGAGACTCAA |
| *P_toxR_* | R | TATTGCTTAGGGGATCAAAGGTAA |
| *P_toxT_* | F | AAATTCTAATTATAAAACGCAAAT |
| *P_toxT_* | R | TGCGTTCTACTCTGAAGATATATA |
| *P_ctxA_* | F | GTGTTCGATACCTTTGCAGCGCAA |
| *P_ctxA_* | R | TTTCATCAGGAGGTCTAGAATCT |
| *P_tcpA_* | F | ATTTCGATCTCCACTCCGGAAATA |
| *P_tcpA_* | R | ATTTATATAACTCCACCATTTGTG |
| Primers for DNaseI footprint | | |
| *P_tcpA_-fam* | F | ATTTCGATCTCCACTCCGGAAATA |
| *P_tcpA_* | R | ATTTATATAACTCCACCATTTGTG |
| Primers for protein purification | | |
| *mlrA* | F | GGAATTCCATATGATGGTTTGTGATGAAAAACGTTAC |
| *mlrA* | R | CCGCTCGAGCTAAGATTGTAGCTGTTGTAGCAC |
| *fnr* | F | CGCGGATCCATGATTTCTGAAAAGCCTGCTG |
| *fnr* | R | CCGCTCGAGTTATTTAGATTCTTTAGATACG |
